# Immunotherapy and associated immune-related adverse events at a large UK centre: a mixed methods study

**DOI:** 10.1186/s12885-020-07215-3

**Published:** 2020-08-10

**Authors:** Liz Jamieson, Martin D. Forster, Kam Zaki, Sanjena Mithra, Heena Alli, Anne O’Connor, Apini Patel, Ian C. K. Wong, Pinkie Chambers

**Affiliations:** 1 UCL School of Pharmacy / UCL/UCLH Centre for Medicines Optimisation Research and Education, 29-39, Brunswick Square, London, WC1N 1AX UK; 2 Department of Oncology, UCL Cancer Institute, 72 Huntley Street, London, WC1 6DD UK; 3 NIHR UCLH Clinical Research Facility, 170 Tottenham Court Road, London, W1T 7HA UK; 4 University College London Hospitals NHS Foundation Trust, 235 Euston Road, London, NW1 2BU UK; 5 Department of Pharmacology and Pharmacy, The University of Hong Kong, L02-56, 2/F, 21 Sassoon Road, Li Ka Shing Faculty of Medicine, Laboratory Block, Faculty of Medicine Building, Hong Kong, China

**Keywords:** Immunotherapy, Immune checkpoint inhibitors, Immune-related adverse events, Lung cancer

## Abstract

**Background:**

The development and rapid uptake of immune checkpoint inhibitors (CPI) has changed the outlook for patients with cancer. However, CPIs have different adverse event (AE) profiles to other systemic therapies, and prompt AE management is essential to assure optimal outcomes. In order to understand what and when adverse events are experienced, reported and managed during CPI treatment, a mixed methods study was conducted, including a case note review of patients who were receiving immunotherapy and semi-structured interviews with patients to understand their experience, management and reporting of AEs after receiving immune CPI treatment.

**Methods:**

This mixed methods study was conducted at a large cancer hospital in the United Kingdom. A case note review identified how and where patients reported AEs. Data relating to patients with lung, bladder, prostate and head & neck cancers who received CPI treatment between 01/04/2015 and 31/07/2018 were extracted from e-prescribing databases and clinical data were included for analysis at a single time point (31 July 2018). Semi-structured interviews were conducted with patients receiving CPI treatment, exploring experience of AEs and reasons for delays in AE reporting and management.

**Results:**

Sixty-two patients were included in the case note review, with 78 AEs being experienced by 36 patients (58%), including one patient experiencing 10 AEs. Serious AEs were experienced by 12 patients (19%) and ten AEs (17%) required oral steroids as treatment. The majority of AEs were reported to clinicians prior to further dosing, although milder AEs were often not addressed until subsequent clinic appointments. Interviews with 13 patients yielded major themes: variability, causality, decision making and impact.

**Conclusion:**

Most CPI-associated AEs are manageable if reported and treated promptly. Both the case note review and interviews found that reporting of non-serious AEs is often left until routine clinic visits, despite impacting patient experience, leaving the opportunity for AEs to be left unreported and implying a potential benefit for real time monitoring. Our study highlights a need to provide patients with reminders around AEs and their timely reporting even when apparently innocuous; patients must understand that AEs can occur at any cycle and even following treatment completion.

## Background

The development and rapid uptake of immunotherapy agents, namely immune checkpoint inhibitors (CPI), is changing the outlook for the treatment of many solid cancers. Long-lasting responses observed in some patients has supported the expansion of their use for a number of malignancies. Additionally, use of combination approaches is growing, with chemotherapy, targeted therapies (such as axitinib for advanced kidney cancer [1], radiotherapy or other novel immune modulating agents. However, caution must temper the excitement of treatment with these agents. Although largely well tolerated, CPIs can generate toxicities including immune-related adverse events (irAEs) [2]. The release of the immune system to activate and expand to fight the cancer may cause inflammation, tissue damage, and even misdirected immune activation against the patient’s own body [3, 4]. Immune checkpoints importantly contribute to the regulation of peripheral tolerance of tissue-specific self-antigens. Therapeutic blockade of these checkpoints may result in a disruption of the balance between tolerance and immunity. This may lead to the development of irAEs although immune regulation is complex and other mechanisms such as epitope spreading may also contribute.

IrAEs mainly involve the gut, skin, endocrine glands, liver, and lung but can potentially affect any tissue [3]. Meta-analyses of clinical trials have shown that 27% of patients treated with CPI inhibitors develop irAEs of some degree, with 6% being severe; a minority experience rare but important side effects that may result in treatment discontinuation [5]; however, the quality of irAE reporting in clinical trials has been shown to be suboptimal [6] and it is likely to be less consistent outside of a clinical trial setting. A more recent systematic review and meta-analysis of 125 studies by Wang et al. (2019) in relation to Treatment-Related Adverse Events of PD-1 and PD-L1 Inhibitors in Clinical Trials found that there was an incidence of at least 66% of patients having 1 irAE or more, with 14% having grade 3 irAEs [7].

The reporting of irAEs is dependent on successful education of patients and carers. Most irAEs experienced due to CPI treatment are mild or moderate and management can be simple if they are recognised early and appropriately treated; with prompt treatment enabling patients to receive the best possible outcomes. However, if not managed quickly and effectively, irAEs can cause long-term harm to patients and are more likely to deem them ineligible for further treatment [8]. It is therefore essential to have effective irAE management pathways in place, to ensure safe delivery and maximise the potential of the agents.

Understanding the reasons for patients’ delay in reporting AEs and subsequent management is fundamental to improving management pathways. Studies in the chemotherapy setting have shown improved outcomes for those reporting chemotherapy toxicity promptly [5]. However, due to the limited experience of wider health professionals around CPI treatment, it is not currently clear why delays in management may be occurring. The use of CPI both in studies and within licensed use has exploded over recent years; much of the available data around irAEs stems from clinical trials, and research is ongoing in the real-world patient populations [9].

The aim of our study was to evaluate all AE reporting and subsequent management in patients receiving CPI treatment. AEs included any AEs that were believed to be treatment-related and this would include irAEs.

## Methods

We conducted a mixed methods study to fully understand the reporting management pathway for all AEs experienced during CPI treatment, consisting of a retrospective case note review and semi-structured interviews with a subset of these patients. The case note review study was designed to evaluate the types and numbers of AEs experienced and to ascertain where reporting took place and where and how management was initiated. The interviews enabled a more detailed understanding of the patient experience in order to understand any reasons for delays in reporting and management.

Patients were included if aged 18 or above and had received CPI (+/− chemotherapy) at UCLH since treatment was available for Lung, Head and Neck, Prostate, and Bladder cancer within late phase studies, early access schemes (EAS) or as standard of care. Patients were excluded if they were receiving treatment within an early phase trial or a placebo-controlled trial in order to ensure that all patients were on a standard CPI. Patients were only included in the interview study if they had received at least 1 cycle of treatment and were receiving or had received CPI treatment within a 6-week window. Further exclusions for the interview element were if patients were unable to make an informed decision to take part or if they did not speak English and a suitable translator was not found.

### Case note review data collection and analysis

Patients were identified through electronic prescribing (EP) systems at UCLH. The following patient details were extracted: age, details of co-morbidities, previous and subsequent treatments, date of first CPI dose, date of death (or the last follow up), number of treatment cycles and deferrals. Two members of the clinical care team (SM and PC) conducted a case note review using electronic hospital records to extract the data, using a structured data extraction form (see supplementary Table 1). This documented the regimen received and the healthcare management of AEs experienced and reported by the patients, along with timing and grade of AEs. Data were recorded electronically and analysed using SPSS version 24 for analysis (SPSS Inc., Chicago, IL, USA). Descriptive statistics were used to summarise the findings.

AEs were graded using the Common Terminology Criteria for Adverse Events (CTCAE) v5.0 [10]. Grade 1 was defined as a mild AE, grade 2 as moderate and grades 3/4 classed as severe. Some patients were taking part in a clinical trial and in these cases trial criteria were used to grade the irAE.

AEs were categorised into organ-specific groups for analysis. We used the coding system of Mekki et al. (2018) to help classify the adverse events [11]. General AEs encompassed non-specific symptoms including fatigue/lethargy, asthenia, pyrexia, and decreased appetite.

### Semi structured interview data collection and analysis

Eligible patients were identified sequentially by clinical pharmacists working in clinical care teams (AP, HA or AOC) through the chemotherapy electronic prescribing records and recruited in the clinic setting.

Information sheets were then given to eligible patients and informed written consent was taken prior to taking part in an interview. Screening logs documented reasons for ineligibility and/ or non-participation of eligible candidates.

Semi-structured interviews were conducted by LJ either on the telephone or face-to-face according to patient preference using an interview guide that was developed specifically for this study by LJ, PC and MDF and has not been published elsewhere (see supplementary file 2). The interview guide included questions relating to patient experience of CPI treatment, irAE reporting, management and impact. With patient permission, interviews were audio-recorded. Due to the nature of the topics discussed in the interview, a safety review was conducted with an independent clinician (KZ) to ensure that all irAEs had been appropriately reported to responsible clinicians. Patients were informed at the start of the interview that this check was necessary for their safety, as the interviewer was non-clinical.

Interview data were transcribed verbatim and identifying data removed. Transcripts were read and coded independently by author LJ and a sample coded by author PC using a general thematic coding methodology. LJ selected all citations that were relevant to the semi-structured questions and coded these into themes using descriptive content analysis. Citations within each theme were further analysed and arranged into subcategories using an inductive process. The team discussed any discrepancies until consensus was reached. The initial coding framework was used to code the subsequent transcripts, and new codes were added as they emerged using a constant comparative technique to compare new and previously collected data to understand emerging themes. Finally, LJ examined the raw data again to ensure that all data were reflected in the coding. The qualitative analysis was facilitated by the use of NVivo software (QSR International [UK] Limited, Southport, UK).

## Results

Table 1 provides an overview of the demographics of patients included in the two elements of the study. The majority of patients in the study were receiving treatment for non-small cell lung cancer (NSCLC), were male, with a median age of 66.

**Table 1:** Overview of Patients included in the study

	Case-note review	Interviews
Total number of Patients	62	13
Male	45	10
Female	17	3
Median age	66 (range 48–81)	
Disease		
Lung	51	11
Prostate	4	0
Bladder	4	1
Head and Neck	2	0
Squamous cell cancer of skin	1	1
Pembrolizumab	55 (11 trial)	10
Pembrolizumab +Docetaxel	2 (1 trial)	
Nivolumab	5 (5 trial)	
Atezolimumab	5	3
Median number of cycles	3 (range 1–32)	9 (range 2–30)

Table 2 shows an overview of AEs from the case note review. The total number of any grade AEs in our case note review was 78, experienced by 36 patients (58%) in 65 episodes, with one patient experiencing 10 AEs. Twenty-six patients (42%) did not experience any documented AEs, 22 of these patients having received fewer than 3 cycles and therefore presumably having little time to experience an AE. Twelve people (19%) experienced at least one serious adverse event. Nine patients had multiple synchronous AEs, where two or three AEs were reported at the same time. The most common any-grade AEs were gastrointestinal (mainly diarrhoea), general (fatigue, fever etc.), respiratory (breathlessness, wheezing) and skin problems (itching, rash). Serious AEs accounted for 21% of the total AEs and included gastrointestinal (multiple GI symptoms), respiratory (acute breathlessness) and rheumatological issues (joint problems).

**Table 2:** Sites of AEs by Grade Following Cycle of Treatment

	Cycles	1	2	3	4	5	6	7	8	9	10	11	12	13	> 13
**Gastrointestinal**															
Diarrhoea	Mild (8)	id1(9) id51(17)		id18(9) id7(32)	id10(20)		id1 (9) id33(9)			id2(24)					
	Moderate (5)					id55(6)	id 36(6)			id4(9)				id60(16)	id60(16)
	Severe (3)			id48(3)	id25(4)			id1 (9)							
Nausea and Vomiting	Mild (1)				id10(20)										
	Moderate (0)														
	Severe (1)	id49(1)													
Mucositis	Mild (2)					id10(20)		id35(7)							
	Moderate (0)														
	Severe (0)														
**Skin**															
Itching	Mild (5)			id3(6) id24(10)	id51(17)					id13(23)	id2(24)				
	Moderate (2)							id8(19)			id8(19)				
	Severe (0)														
Rash	Mild (2)	id6(7)							id10(20)						
	Moderate (0)														
	Severe (0)														
**Cardiovascular**															
Chest pain and discomfort	Mild (0)														
	Moderate (1)					id1(9)									
	Severe (0)														
**Eye**															
Visual disturbances	Mild (1)	id2(24)													
	Moderate (0)														
	Severe (0)														
Conjunctival haemorrhage	Mild (1)	id5(11)													
	Moderate (0)														
	Severe (0)														
**General**															
Fatigue	Mild (6)			id2(24) id3(6) id54(5) id7(32)		id10(20)								id2(24)	
	Moderate (3)	id56(6)	id12(6)								id13(23)				
	Severe (0)														
Dizziness	Mild (0)														
	Moderate (1)	id16(3)													
	Severe (0)														
Nail Changes	Mild (1)			id37(5)											
	Moderate (0)														
	Severe (0)														
Other	Mild (1)			id54(5)											
	Moderate (0)														
	Severe (1)	id58(1)													
Fever	Mild (2)	id54(5)	id59(2)												
	Moderate (1)	id27(1)													
	Severe (2)				id4(9) id25(4)										
**Endocrine**															
Syndrome of Inappropriate Antidiuretic Hormone (SIADH)	Mild (0)														
	Moderate (1)	id56(6)													
	Severe (0)														
Hypothyroidism	Mild (1)													id2(24)	
	Moderate (5)				id13(23) id51(17)			id7(32)		id14(19) id7(32)					
	Severe (0)														
Raised blood glucose	Mild (1)	id5(11)													
	Moderate (0)														
	Severe (0)														
**Respiratory**															
Cough	Mild (1)	id32(1)													
	Moderate (0)														
	Severe (0)														
Shortness of Breath	Mild (4)	id6(7)		id2(24)											id2(24)
	Moderate (4)		id9(17)		id55(6)			id7(32)						id2(24)	
	Severe (2)	id15(1) id 36(6)													
**Renal**															
Renal function	Mild (1)	id6(7)													
	Moderate (0)														
	Severe (0)														
**Rheumatological**															
Joint Pain	Mild (3)	id17(2)		id56(6)					id51(17)						
	Moderate (1)	id9(17)													
	Severe (2)	id11(17) id18(9)													
**Musculoskeletal**															
Bone Pain	Mild (0)														
	Moderate (2)	id34(3)								id51(17)					
	Severe (0)														
**Hepatic**															
Liver function tests deranged	Mild (0)														
	Moderate (1)													id60(16)	
	Severe (0)														
	TOTAL Mild (40) Moderate (27) Severe (11) *n* = 78														

*NB* Following patient id bracket = number of cycles received

### Management of AEs

From the case note review, the majority of mild or moderate AEs were reported to a doctor (oncologist) at a scheduled pre-treatment clinic visit as shown in Table 3. Ten AEs (experienced by 10 patients) required oral steroids as treatment. Steroids were prescribed in accordance with the European Society of Medical Oncology guidelines [12]. Specifically, the AEs that led to steroid initiation were diarrhoea (*n* = 6), derangement in liver function (*n* = 1), pain (*n* = 2), and shortness of breath (n = 1). Although systemic steroids are primarily used in the management of moderate-severe grade AEs, one patient with a mild but persistent AE also required systemic steroids. The time to initiate steroid was dependent both on the time of AE reporting and severity of the AE. Seven of the ten patients given steroids had either presented via the Emergency Department (ED) or required a hospital admission. Overall, eight AEs resulted in a hospital admission and 8 in attendance at the ED, the patient either going straight there or advised to attend ED by helpline staff and one patient was admitted to hospital when AEs were reported in clinic.

**Table 3:** Method of reporting immune related adverse events by patients

How reported	Grade of AE			
	Mild	Moderate	Severe	
24 h help line	1	3	6	10
To doctor in clinic	27	15	2	44
To nurse in clinic	1	0	0	1
To emergency department	0	2	2	4
Helpline, then emergency department	1	1	1	3
Other	0	1	0	1
Not reported (n = 1)				1
Picked up at monitoring	0	1	0	(1)
	30	23	11	65

**Fig. 1:**
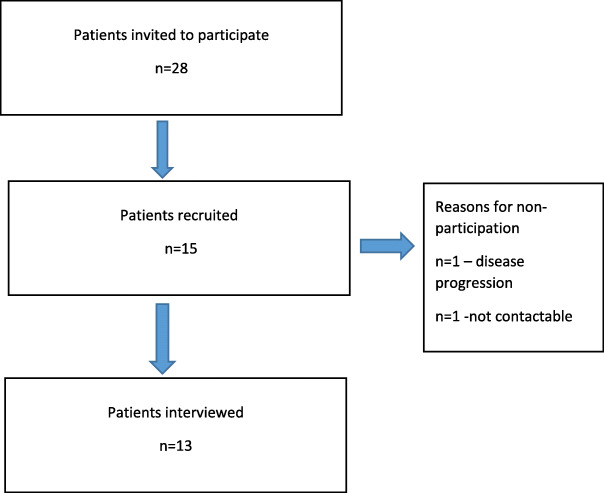
Flow diagram of Patients Recruited to the Interview Study

**Fig. 2:**
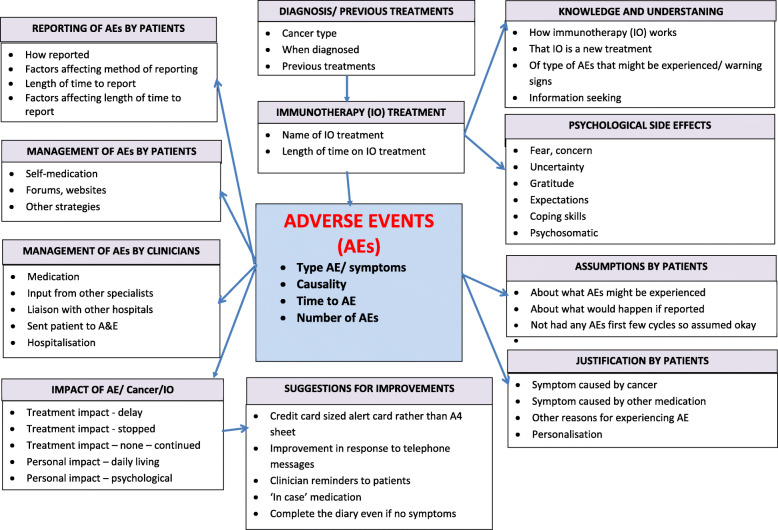
Thematic model of Patient Experiences with irAEs

Figure 1 depicts the recruitment of patients for interviews. In total 13 patients participated in the interview study and Fig. 2 shows the initial model that was developed through thematic analysis of the interview data (Supplementary file 2 (tables 2–5) contain supporting quotes for the development of themes from the interview data).

Some of these initial themes were subsumed under overarching themes: in relation to diagnosis, reporting and management of irAEs, variability, causality, decision-making and impact.

Variability was found across the following

- Patient histories (prior treatments, co-existing conditions and other medications)
- Knowledge and understanding of immunotherapy and what constitutes an adverse event (how CPI work, realisation and knowledge that CPIs are a new, expensive treatment, understanding of the information seeking
- Type and number of irAEs experienced
- Time to irAE

Patients receiving CPI treatment varied significantly in their medical histories, personal experiences and other co-morbidities (e.g. diabetes, stroke, COPD, arthritis), all of which contributed to their experience and management of AEs. This variability was also seen in their knowledge and understanding of AEs, despite all receiving treatment at the same hospital. Most patients knew the name of their treatment, even if they were unable to pronounce it. Some referred to it by the trade name, e.g. Tecentriq. Patients had mixed understanding of *how* CPI works. However, all understood that immunotherapy is a new treatment and there is a need for further research as to its effectiveness. Most people had some recollection of types of AEs to look out for, but several people had sought out further information, either from friends or family or from the internet. One man had obtained additional leaflets from the MacMillan Cancer Centre Information point.

The number of cycles of immunotherapy treatment that the participants had received ranged from 2 to 30 with a median of 9.

There was some confusion about what constituted an AE. The impact or potential impact of treatment was a factor that influenced reporting. All patients who had experienced prior chemotherapy or radiotherapy described feeling physically much better very soon after commencing the CPI treatment. There was a wide range of AEs discussed by participants that varied in presentation and onset. Six patients had not experienced any AEs at all, whereas others had quite severe symptoms such as breathlessness, skin problems, and diarrhoea.

### Causality

- Justification of symptoms by patients (symptom(s) related to cancer, symptom(s) legacy from prior treatment, e.g. chemotherapy or radiotherapy, symptom(s) related to other medication and psychological side effects, e.g. tiredness, anxiety, psychosomatic symptoms
- Personalisation – “normalisation” of symptoms by patients
- Uncertainty on the part of clinicians

Understanding the causality of symptoms by both patients and healthcare professionals came through as a theme influencing reporting and management. Patients ignored symptoms such as tiredness, flaky nails, mild aches and pains; one or two people denied experience of any AEs and then described potential AEs on further questioning. Determination of causality was often a process of elimination whereby patients tried to reason whether a symptom was likely to be caused by the cancer itself, or the treatment: examples include tiredness and breathlessness, especially in patients with NSCLC. Sometimes patients felt that the symptoms were caused by other medication, e.g. sleepiness or diarrhoea, or by other factors such as having eaten something, being abroad, or diarrhoea causing fatigue. Patients used personalisation, i.e. what is ‘usual’ or ‘normal’ for them to judge the type and severity of any AE using that as a benchmark in this appraisal process. A few patients also believed that AEs would only occur when commencing treatment (within the first few cycles) and seemed unaware that occurrence could be at any point.

*“It all started after the 5*^*th*^ *cycle. I started to get the diarrhoea and I never actually gave it a thought that it was anything to do with the treatment…The thing about the diarrhoea was it didn’t wake me up so I went, I was going, say, 5 or 6 times during the day but not the night, I wasn’t feeling sick, I had no stomach cramps. No nausea, my appetite was okay so I thought this is strange”. [0004]*

*“I never actually gave it a thought that it (the diarrhoea) was anything to do with the treatment.” [0003]*

Clinicians and patients alike were often unsure whether an AE was related to the CPI treatment or not, mainly due to multiple possible confounding factors, and would frequently choose to monitor symptoms initially. This was seen in the case note review when patients had gaps in the treatment schedule from commencing steroid.

*“… they thought it might have been a clot on the PICC line, but they did a scan and that was all fine, so they eliminated that, they eliminated any sort of blood clots around the line or anything, and came to the conclusion that it probably was the drug, that was like a reaction to it.” [0007].*

### Decision making

- Reporting a potential irAE by patients (how reported, factors affecting method of reporting, length of time to report, factors affecting length of time to report)
- Management of a potential irAE by patients (self-medication, forums and websites, other strategies)
- Management of a potential irAE by clinicians (medication, input from other specialists/ liaising with hospitals, send patient to A&E, hospitalisation)

A theme of considered decision making by the patient and health professionals contacted was frequently found in the interviews. Some participants reported AEs quickly via the hospital helpline whereas some patients waited to discuss symptoms at the next clinic appointment.

*.. “so this was a period of days before your next clinic?”[interviewer] “It was a week”. [0002]*

Most patients preferred to wait until their next clinic appointment to discuss any symptoms with their clinician; this was observed in both the case note review and interviews. Participants described how they viewed their oncologist as the expert but also, they often did not believe their symptom(s) to be serious. Some patients were reluctant to call hospital helplines not wanting to be a nuisance and recognising that clinic staff were very busy.

*“I did that a couple of times, had to phone out-of-hours, and in general, they're going, "Oh, I don't know, can you phone back in the morning". [0002]*

*“I actually virtually crashed in the clinic. And they looked after me until they could get me round to A&E (Accident & Emergency/ ED), who looked after me some more while they all decided what was wrong with me, a big committee of doctors surrounding me in resus (resuscitation) in A&E, and eventually at about eleven o'clock at night they threw me in an ambulance and shot me off to hospital”. [0002]*

Some patients tried to manage their irAE by self-medication using their community pharmacies, e.g. E45 cream for skin problems, Imodium for diarrhoea, naproxen for arthritis, paracetamol for pain. Advice was gained from forums and websites involving others experiencing similar symptoms. Patients additionally described discussions with family members and knowledgeable friends.

Management of AEs by clinicians included giving medication for symptoms, e.g., antibiotics, steroids, or obtaining input from specialists such as rheumatology and haematology or by liaising with other hospitals. Several patients spoke of being directed to A&E by the clinic staff or, in one or two cases, hospitalised.

### Impact

- Of CPI treatment (Physical, Psychological)
  - ° Of irAE (Physical, Psychological)

All patients who had experienced prior chemotherapy or radiotherapy described feeling physically better very soon after commencing the CPI treatment.

Apart from the physical impact of the disease and immunotherapy treatment, patients also described psychological side effects, some of which were related to the CPI treatment itself, for example, their own expectations and the significance of the treatment being their last chance at extending their life. The gratitude felt by patients receiving treatment meant that they did not like to complain. Other patients who were approaching the end of the two-year period on CPI treatment spoke about their worry about what, if anything, could or would be offered next and whether the cancer would return. Many patients experienced cyclical anxiety and spoke about the fear that they experienced prior to having a scan and attending clinic for the result, and the huge disappointment they experienced if the scan results were not favourable or relief if it was a positive outcome.

*…“If they say, at some point, "You can’t have it any more because it’s doing you more harm than good", then quite what plan B will be, I don't know, and I don’t really want to think about plan B until plan A is no longer an option” [0002]*

## Discussion

Our case note review findings of 58% of patients experiencing any grade AEs and 19% serious AEs is similar to the findings of the Keynote-042 trial involving pembrolizumab, where 63% experienced any grade AE and 18% events of grade 3 or worse severity, mainly pneumonitis, severe skin reactions, and hepatitis [13].

This study, which supplements a case note review with detailed patient interviews, helps to understand where interventions should be targeted to improve the management pathways for irAEs. We found from our case note review that many ‘minor’ AEs are left unreported and clinic assessments prior to treatment provide patients the opportunity to relay Information. However, it is unclear whether these AEs would have ever been reported without prompting, or how many other AEs may often be left unreported. Findings from this mixed methods study were that patients were not accessing prompt management by delaying seeking help for their AEs; especially if they inferred them as either trivial or non-treatment related.

In many cases the timing of AE management may be insignificant; however, from our case note review we found examples of cases where minor AEs escalated to major ones and, in turn, required hospital admissions. This is similar to findings by Basch et al. [14] where symptom reporting in real time, in patients receiving chemotherapy rather than immunotherapy, led to reduced hospital admissions. Approximately one sixth of patients attended the emergency departments (ED), either directly or following guidance provided by the emergency helpline, because of an AE, highlighting the potential burden that these agents may add to the health system as their use expands. This figure may reduce with better management strategies, including prompt reporting. The current and future economic impact of this additional caseload on emergency departments is unknown.

In observing the variety of AEs seen, we noted the role of multiple specialisms, where input is essential to assure patient safety. Specialisms such as rheumatology and endocrinology are commonly called upon to provide specialist input. A recent cross-sectional study [15] using an algorithm-driven approach to characterize immune-related adverse events found that the diagnosis and characterization of immune-related adverse events are challenging. There was poor concordance of interrater agreement in the occurrence, severity, and timing of 8 common immune-related adverse events. Discordance was associated with longer durations of therapy and higher comorbidity burden in patients.

Interestingly, the themes we found from the interview data were remarkably concordant with the work of Scott et al. [16] in the early diagnosis of cancer setting. This model, known as Walter’s model, considers the contribution of patients, providers/system and disease factors to four intervals: (Symptom) Appraisal, Help-seeking, Diagnostic and Pre-treatment. It is presented mainly as a linear sequence leading to diagnosis but acknowledges the possibility that patients move back and forth between intervals in consultation with healthcare professionals (HCPs). The theme of variability would feature in contributing factors; causality would represent the appraisal process and decision-making could be considered to be the appraisal and help seeking stage. We found there were delays by patients in appraisal as well as help-seeking. We believe this is the first study where AE management in cancer patients has been found to be remarkably similar to pathways for diagnosis of cancer, supporting use of this model to guide understanding around delays to treatment as others have done in the early diagnosis setting.

An area where the Walters model could be further tailored to toxicity management for newer treatments is in understanding the gratitude that patients feel when they receive new treatments. This finding came across many times in the interviews and should be explored further as the anxiety around treatment cessation may contribute to delaying the appraisal and help seeking phases. In addition, the complexity of patients’ histories, diseases and concurrent medications can mean clinicians and patients experience difficulties in distinguishing disease or co-morbidity-related symptoms to those of an irAE and this may lead to delays in management.

Psychological ‘side effects’ such as anxiety and psychosomatic symptoms in relation to the disease itself cannot be ignored. This has similarities to the uncertainty described in a study examining the experiences of patients with metastatic melanoma undergoing pembrolizumab immunotherapy [17]. This observed that metastatic melanoma patients coped within a spectrum of uncertainty increasing before and during cycles of treatment, monitoring and investigations, then subsiding between, allowing patients to re-engage in their lives.

### Suggestions for improving follow up

The most useful area for our organisation was the acknowledgement that delayed reporting is not only a delay in accessing advice but was also related to the patient’s own lack of recognition that a symptom is actually related to treatment. This appraisal process by patients can be improved through more timely information and other educational support. We believe real-time reporting could be valuable but cannot be relied on solely. An eHealth intervention based on questions from the PRO-Common Terminology Criteria for Adverse Events (CTCAE) library was used and tested in a randomized clinical trial with patients receiving immunotherapy for malignant melanoma and clinicians at a hospital in Denmark [18]. The primary objective of this study was to examine patients’ and clinicians’ experiences with an eHealth intervention for weekly monitoring of side effects during treatment with immunotherapy. Overall, satisfaction with the eHealth intervention was high among patients and their treating clinicians. The tool was easy to use and contributed to greater symptom awareness and patient involvement.

As a result of the SARS-CoV-2 pandemic, patients are not being seen as frequently in clinic, and therefore it is even more important to assess regularly in the form of telephone or video consultations and we suggest that clinicians consider the following areas during these sessions:

- Detailed probing of even minor symptoms
- Attempt to understand what patients consider ‘normal’ for them or may be attributing to other things, such as prior treatments or side effects of other medications and co-morbidities
- Remind patients regularly that AEs can occur at any point during or even after treatment.
- Remind patients to report or seek advice as soon as possible and the benefits of treating early
- Ask patients what medicines or creams they might have bought from pharmacies, including over the counter medicines or
- Ask patients whether they have sought information on immunotherapy or advice about a particular side effect either on-line or from a colleague or friend/relative.

### Strengths and limitations

Our study was unique in that patient interviews explored reasons for delayed AE reporting, and we were able to find themes that might help with the development of interventions. We only examined data from one large single centre, which may limit the generalisability of some findings. We also acknowledge that the data extraction form needs to be tried out in larger studies in a variety of settings and further modified. However, we believe that many of the findings will be relevant to other centres and that this small study is an important step to obtaining patient outcome data in this new field. Additionally, many patients were not eligible to take part in our study as they were receiving treatment within a clinical trial, which limited the patient numbers. The retrospective nature of the case note review made it difficult to confirm the accuracy of reported grading. Despite thematic saturation being achieved we accept that our sample size was limited; however, our study complements findings from similar studies in this area, as well as broader toxicity management evaluations, which justifies the transferability of our findings. In fact, a recently published study [19] examined patients’ experiences with immune checkpoint modulators, recruited from one organisation in Toronto, Canada, with a focus on their side effects and how these impacted on their daily life. They identified eight themes, characterising the complexity of these patients’ lived experiences: major categories of side effects experienced and how they impacted patient well-being; the heterogeneous nature of side effects experienced, all of which were reflected in our category of ‘variability’; living with uncertainty; reframing the meaning and severity of SEs; focus on survival, hope, and being positive; acceptance and adaptation; feeling supported; and faith in medical innovation, which correspond to our psychological impact categories.

## Conclusions and recommendations for further research

This mixed methods study highlights key factors important to target in terms of prompt AE management. The most salient area from our work was that patients need to firstly understand that they are experiencing an AE and then promptly seek help from the relevant health care professional in real time. Implementation of existing research into real time monitoring could enable prompt management with fewer hospital admissions for this expanding group of patients.

## Supplementary information

**Additional file 1.** Data extraction form for case note review.

**Additional file 2.** Interview guide for semi-structured interviews and tables 2–5 themes of variability, causality, decision making and impact.

## Data Availability

The authors will make data available on request.

## Abbreviations

AE: Adverse Event
CPI: Checkpoint inhibitor
irAE: immune related adverse event
ED: Emergency department
UCLH: University College London Hospitals NHS Foundation Trust

## References

[1] Rini BI, Powles T, Chen M, Puhlmann M, Atkins MB (2017). Phase 3 KEYNOTE-426 trial: Pembrolizumab (pembro) plus axitinib versus sunitinib alone in treatment-naive advanced/metastatic renal cell carcinoma (mRCC). J Clin Oncol.

[2] Ramos-Casals M, Brahmer JR, Callahan MK, Flores-Chavez A, Keegan N, Khamashta MA, Lambotte O, Mariette X, Prat A, Suarez-Almazor ME (2020). Immune-related adverse events of checkpoint inhibitors. Nat Rev Dis Primers.

[3] Michot JM, Bigenwald C, Champiat S, Collins M, Carbonnel F, Postel-Vinay S, Berdelou A, Varga A, Bahleda R, Hollebecque A (2016). Immune-related adverse events with immune checkpoint blockade: a comprehensive review. Eur J Cancer.

[4] Spain L, Diem S, Larkin J (2016). Management of toxicities of immune checkpoint inhibitors. Cancer Treat Rev.

[5] Wang PF, Chen Y, Song SY, Wang TJ, Ji WJ, Li SW, Liu N, Yan CX (2017). Immune-related adverse events associated with anti-PD-1/PD-L1 treatment for malignancies: a meta-analysis. Front Pharmacol.

[6] Bertrand A, Kostine M, Barnetche T, Truchetet ME, Schaeverbeke T (2015). Immune related adverse events associated with anti-CTLA-4 antibodies: systematic review and meta-analysis. BMC Med.

[7] Wang Y, Zhou S, Yang F, Qi X, Wang X, Guan X, Shen C, Duma N, Vera Aguilera J, Chintakuntlawar A (2019). Treatment-related adverse events of PD-1 and PD-L1 inhibitors in clinical trials: a systematic review and meta-analysis. JAMA Oncol.

[8] Voskens CJ, Goldinger SM, Loquai C, Robert C, Kaehler KC, Berking C, Bergmann T, Bockmeyer CL, Eigentler T, Fluck M (2013). The price of tumor control: an analysis of rare side effects of anti-CTLA-4 therapy in metastatic melanoma from the ipilimumab network. PLoS One.

[9] Cavaille F, Peretti M, Garcia ME, Giorgi R, Ausias N, Vanelle P, Barlesi F, Montana M. Real-world efficacy and safety of pembrolizumab in patients with non-small cell lung cancer: a retrospective observational study. Tumori. 2020;300891620926244. 10.1177/0300891620926244. 10.1177/0300891620926244 32458769

[10] Common Terminology Criteria for Adverse Events (CTCAE) version 5 2017 [https://ctep.cancer.gov/protocolDevelopment/electronic_applications/ctc.htm#ctc_50]. Accessed 1 Jan 2019.

[11] Mekki A, Dercle L, Lichtenstein P, Marabelle A, Michot JM, Lambotte O, Le Pavec J, De Martin E, Balleyguier C, Champiat S (2018). Detection of immune-related adverse events by medical imaging in patients treated with anti-programmed cell death 1. Eur J Cancer.

[12] Haanen J, Carbonnel F, Robert C, Kerr KM, Peters S, Larkin J, Jordan K, Committee EG (2017). Management of toxicities from immunotherapy: ESMO Clinical Practice Guidelines for diagnosis, treatment and follow-up. Ann Oncol.

[13] Mok TSK, Wu Y-L, Kudaba I, Kowalski DM, Cho BC, Turna HZ, Castro G, Srimuninnimit V, Laktionov KK, Bondarenko I (2019). Pembrolizumab versus chemotherapy for previously untreated, PD-L1-expressing, locally advanced or metastatic non-small-cell lung cancer (KEYNOTE-042): a randomised, open-label, controlled, phase 3 trial. Lancet.

[14] Basch E, Deal AM, Kris MG, Scher HI, Hudis CA, Sabbatini P, Rogak L, Bennett AV, Dueck AC, Atkinson TM (2016). Symptom monitoring with patient-reported outcomes during routine Cancer treatment: a randomized controlled trial. J Clin Oncol.

[15] Hsiehchen D, Watters MK, Lu R, Xie Y, Gerber DE (2019). Variation in the assessment of immune-related adverse event occurrence, grade, and timing in patients receiving immune checkpoint inhibitors. JAMA Netw Open.

[16] Scott SE, Walter FM, Webster A, Sutton S, Emery J (2013). The model of pathways to treatment: conceptualization and integration with existing theory. Br J Health Psychol.

[17] So AC, Board RE (2018). Real-world experience with pembrolizumab toxicities in advanced melanoma patients: a single-center experience in the UK. Melanoma Manag.

[18] Tolstrup LK, Pappot H, Bastholt L, Zwisler AD, Dieperink KB (2020). Patient-reported outcomes during immunotherapy for metastatic melanoma: mixed methods study of Patients' and Clinicians' experiences. J Med Internet Res.

[19] Ala-Leppilampi K, Baker NA, McKillop C, Butler MO, Siu LL, Spreafico A, Abdul Razak AR, Joshua AM, Hogg D, Bedard PL, et al. Cancer patients' experiences with immune checkpoint modulators: a qualitative study. Cancer Med. 2020;9(9):3015-22. 10.1002/cam4.2940 PMC7196048 32119767

