# Supplementary material for: Immunotherapy and associated immune-related adverse events at a large UK centre: a mixed methods study
Source: BMC Cancer. 2020 Aug 10;20:743. doi: 10.1186/s12885-020-07215-3 (PMC7416581; doi:10.1186/s12885-020-07215-3)
Supplement: Supplementary file 1 — Additional file 1. Data extraction form for case note review. [file 12885_2020_7215_MOESM1_ESM.docx]

Supplementary File 1**:** Data extraction form for the case note review and tables of major themes with supporting quotes from the interview study

**Supplementary Table 1.DATA EXTRACTION form**

**Immunotherapy Related Adverse Events – The Patient Experience**

| ID |  |  |
| --- | --- | --- |
| Age (years) |  |  |
| Gender | 1. Male 2. Female |  |
| Type of cancer | 1. Lung 2. Bladder 3. Prostate 4. Head & Neck 5. Melanoma |  |
| Stage of cancer  Grade of cancer | I, II, III, IV |  |
| Co-morbidities | Please list all |  |
| Taking part in clinical trial | 1. Yes [Name] 2. No |  |
| Date of start of immunotherapy treatment |  |  |
| Length of time on immunotherapy treatment |  |  |
| Number of cycles received to date (including current cycle) |  |  |
| Type of immunotherapy treatment | 1. Pembrolizumab 2. Ipilimumab 3. Nivolumab 4. Combination therapy (specify), e.g. Pembro/ Docetaxel 5. Clinical trials 6. Other |  |
| Line of therapy |  |  |
| Immunotherapy stopped | 1. Yes 2. No (ongoing) |  |
| Date of cessation of immunotherapy treatment (if applicable) |  |  |

**Adverse event 1**

| Date adverse effect 1 reported (if no adverse effects reported please specify NONE REPORTED) |  |  |
| --- | --- | --- |
| Length of time after start of treatment until adverse effect 1 | Weeks/months or between cycles |  |
| Type of adverse effect 1 | List main symptoms |  |
| Grade of adverse effect 1 | 1. Mild 2. Moderate 3. Severe |  |
| How adverse effect 1 reported | 1. Not reported 2. Helpline 3. To doctor in clinic 4. To nurse on phone or in person 5. Other (give details) |  |
| Self-help treatment prior to reporting | 1. Over the counter medicine 2. Over the counter skin cream 3. Dietary changes 4. Herbal medicines 5. GP treatment (specify) 6. Other (please specify) 7. None |  |
| Adverse effect 1 require admission (Y/N) | 1. Yes – A&E 2. Yes - hospitalisation 3. No |  |
| Adverse effect 1 require stopping treatment (Y/N) | 1. Yes 2. No |  |
| Treatment given for adverse effect 1 | 1. None – monitor 2. Steroid treatment 3. Hospitalisation 4. Medication (pls specify) 5. Other (please specify) |  |
| Time to receive steroid | 1. From start of treatment  2. From reporting AE |  |
| Impact of adverse effect 1 | 1. Dose delay (number of days) 2. Dose reduction (% dose) 3. Treatment stopped |  |
|  |  |  |
