# Supplementary material for: Immunotherapy and associated immune-related adverse events at a large UK centre: a mixed methods study
Source: BMC Cancer. 2020 Aug 10;20:743. doi: 10.1186/s12885-020-07215-3 (PMC7416581; doi:10.1186/s12885-020-07215-3)
Supplement: Supplementary file 2 — Additional file 2. Interview guide for semi-structured interviews and tables 2–5 themes of variability, causality, decision making and impact. [file 12885_2020_7215_MOESM2_ESM.docx]

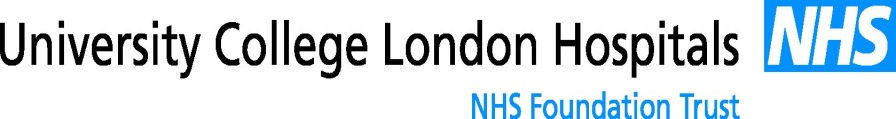


## Supplementary File 2: INTERVIEW GUIDE

**Immunotherapy Related Adverse Events – The Patient Experience**

- **Story of illness and treatment**

Can you tell me a little bit about how you came to have immunotherapy and how long you have been having it?

Prior to starting immunotherapy:

- Did anyone explain about the potential side effects to you?
- Who explained about the potential side effects?
- What did they tell you about how to manage the potential side effects?
- **What side effects have you experienced on this treatment**?
  - Tell me about the side effect. [Description of symptoms, etc.]
- How soon did you experience the side effect after commencing treatment?
- Was this the first time you have experienced a side effect from the treatment?
- **Reporting of adverse event**

I would like you to tell me a bit more about the circumstances that led up to you reporting or managing the side effect yourself. Taking each side effect in turn:

- Did you tell anyone or seek help from anyone to manage this side-effect?
  - What did they say?
- How long did you wait before seeking help?
- What made you decide to report this side effect to the hospital?
- How easy did you find the reporting process you used?
- What made you decide to use the method you used to report it [helpline/ wait until next clinic appointment etc./ GP]
- How easy did you find it to describe the side effect you experienced?
- What made you think the symptom(s) were related to the treatment?
  - How certain were you?
- Why did you not report if [if applicable]?
- How did you manage it?

- **Outcome of reporting of adverse event**
  - How did the person dealing with it manage your side effect? What advice did they give you?
    - Was this satisfactory to you?
  - Was the treatment stopped?
- Was this because of the side-effect?
- If yes, who made the decision to stop the immunotherapy?
- **Impact**
  - What has been the impact on you [emotionally, physically]?
- Of immunotherapy
- Of any adverse effects you have experienced
- Of treatment for adverse effect
- **Learning from experience**
- If you experienced another side-effect would you do anything differently?
- Is there anything we can do to improve our reporting system, patient information or management of side effects?

**Supplementary table 2. Quotes relating to Major theme -Variability**

|  |  |  |
| --- | --- | --- |
| **Patient histories** | Prior treatments | “I had, originally I had chemotherapy, I had three sessions, which didn't work, I got more tumours and other things were going wrong, so it didn't stop the spread of the cancer and it made me violently ill”. (0007) |
|  |  | “Yeah, he had the chemo first, six cycles. ..then they gave him radiotherapy for nine times we had that”. (0013) |
|  | Other medications | “I’m taking Levothyroxine. Because of hypothyroid*. [Are you taking any other medication regularly ...?]*  Yes, I'm taking Metformin,... *[Is that for diabetes?]* Yeah, which is better now because I've lost weight. I'm taking Levothyroxine. I'm taking Spironolactone for the fluid. Er… Omeprazole. And Calciferol, vitamin D. I also take Fluoxetine, one a day, but I'm not very regular on it”. (0012) |
| **Knowledge and Understanding of immunotherapy and AEs** | How IO works | .. “they explained the idea of the treatment is that cancer cells use immune, part of the immune system to hide themselves from, sorry, they use certain enzymes, whatever, to hide themselves from the immune system and that this treatment helps the cancer, the immune cells find those cancerous cells”. (0003) |
|  |  | .”and Dr X did explain that immunology works slightly slower than the chemotherapy, that the progress is slower but surer than the chemotherapy” (0010) |
|  | Recognition that IO is a new, expensive treatment | “Well, they said it was new, fairly sort of experimental, I had read up on it, that it was used for the melanoma, wasn't it, to start with, yeah, so they said they would, because of the molecule matching and everything, they said they would try it and see what happens, and it's worked amazingly”. (0007) |
|  |  | *“When they told you about the immunotherapy treatment, can you remember, I know it's two years ago now for you, but can you remember what you were told about it, did you know what to expect?*  They had good results on it, didn’t they, cost a lot of money. We'd actually seen it in telly, you know, about it. *[Oh, had you].* So we knew America were getting good results from it” (0008) |
|  |  | “Immunotherapy, which is this new, very expensive but very effective, usually, treatment, which basically gives one’s own immune system a bit of a kick up the bottom”. (0009) |
|  | Understanding of type of AEs that might be experienced/ warning signs | “Well, for instance, they reckon you had to watch your temperature, if you get a fever over a certain, I think it was 36 or whatever it was, you’ve got to contact the hospital immediately”. (0001) |
|  |  | “..it was explained to me that side-effects whilst not being common, when they are discovered are quite severe. But generally the medicine’s well tolerated and it was also explained to me very explicitly that this is a very new drug and that a lot of the things that may be, that may occur or may happen haven’t been documented or reported enough so that we can tell you that. So I was fairly well informed….any kind of infection or fever”. (0003) |
|  |  | “Yes, that it could trigger the immune system to attack its own cells, that was the worst”. (0012) |
|  | Assumptions about timing of irAEs | “So the big issue for me side-effects has been the diarrhoea. I suppose my main disappointment and I can’t really blame anybody because I should have thought of it as well was that having had, say, the 4 cycles and they’re saying oh you’re tolerating it well, there’s no problems, no problem, it never crossed my mind that the diarrhoea could have been triggered by that initially and I suppose nobody reminded me”. (0004) |
|  | Information seeking | ”The possible side-effects were sort of explained but I’d actually, before it all started I did call in at the Macmillan Centre and they gave me some Cancer Research UK information on it and from that I found things like feeling or being sick, pain or swelling of joints, diarrhoea, skin changes, fatigue, so I was aware of that and we did discuss that”. (0004) |
| **IrAEs experienced and time to irAE** | I have been good, actually, nothing wrong. [*Right. Have you had any side-effects at all?]* Nothing”[after 9 cycles]. (0013)  *“Has he experienced anything else at all? I mean I know you’ve told me he hasn’t but I’d like to hear him say that he’s felt okay too***?** *[Translates]* No, nothing”. [after 10 cycles] (0010) | |
|  | “I had the first round of treatment and I had a reaction to it, which came very close to killing me. *[Okay, and what sort of symptoms did you have?]* The symptoms I had was, I had absolutely no appetite at all, I was very, very, very short of breath, I mean, I couldn't walk more than about six or eight feet, I was in a dreadful state…I actually virtually crashed in the Clinic. …..it would have been cycle twenty, I think, somewhere around there, I went along, had cycle twenty as normal and after a few days I started getting a range of symptoms, one at a time. *[What symptoms did you have at this point?]* Well, again, this was swollen knees, shortness of breath, you know, no energy at all because I hadn't been eating all that much, and the journey from home to the clinic had really exhausted me, really. (0002) | |
|  | “It all started after the 5th cycle.. I started to get the diarrhoea and I never actually gave it a thought that it was anything to do with the treatment…The thing about the diarrhoea was it didn’t wake me up so I went, I was going, say, 5 or 6 times during the day but not the night, I wasn’t feeling sick, I had no stomach cramps. No nausea, my appetite was okay so I thought this is strange”. (0004) | |
|  | “This fatigue had been quite intense on some occasions, where I've been in bed all day and I've slept, on and off, pretty much, you know….*[Yes, so the fatigue had started anyway because of the cancer?]* Yeah, there was some fatigue there before, but … it definitely got worse when I was initially on the immunotherapy”. (0005) | |
|  | “The first two infusions went extremely well, I had really hardly, you know, something, you know, sort of a little minor, you know, the next morning not quite a hangover but you felt oh something’s happened *[laughs]*, but that was really…the third infusion at the end of May, within 48 hours I developed an extensive rash over the whole body which looked as if I had a serious case of measles”. [0006) | |
|  | ”I did have a couple of side-effects at the very beginning, which they put down to the new drug, because...[*When you say at the very beginning, I mean, after the first infusion...?]* Yes, after the first one, because in the July I had the PICC line put in because it was so difficult to get the carrier in, and so I had the first one in, I think it was the end of July, and my arm swelled up, my right arm swelled up, and that's where the PICC line is, so it was quite painful, actually, and it all swelled up and I couldn't get my watch on and things. And they thought it might have been a clot on the PICC line, but they did a scan and that was all fine, so they eliminated that, they eliminated any sort of blood clots around the line or anything, and came to the conclusion that it probably was the drug, that was like a reaction to it.” (0007). | |
|  | *“Up to this point had you had any bad side-effects from the Pembro****?*** No. *[Nothing at all?]* No, no. *I can't think of anything. [So you went for a year and you didn't experience anything?]*  No, not really, I didn’t, never, actually. The only, out of all the years, I suppose I've got a blocked nose and I can't breathe properly*. [Is that something you've had before this?]* No. *[So this breathing thing, have you mentioned that to the doctor?]* Yeah”. (0008) | |
|  | “And it’s been alright. I was a bit, I don’t know, I think my stomach was a bit funny at first. I was getting like diarrhoea and that. *[I’m asking you to remember quite a while back but how soon after starting it?]* I found that if I was going out for a pint or if I had curries or stuff like that, and coffee. *[And coffee? Okay.]* Yeah. *[So alcohol or caffeine you think?]* Caffeine and alcohol. So as I say I cut down on both”. (0011) | |
|  | “And after the third cycle I've got some issues with my bowels. *[Okay, when did this start?]* That started er… like ten days after the third cycle? *[Cycle three?]* Cycle three. *[Okay]***.** Probably, I probably aggravated it with eating a lot of fibre, but, because I've diverticulitis, so...*[Right, so you have to be careful anyway, do you?]* Yeah, but I wasn’t, I ate a lot of cabbage and all that, so I've got pain and I've got blood in my stools. I'm going to ask them for antibiotics for some... *[And have you mentioned this to anybody?]***…**No, no. *[But you're going today?]* Yes, today, and then my GP in the afternoon. But it got worse during the weekend. *..[Is it the pain or is it diarrhoea?]* No, it's painful, more like gas. *[And is it like your normal diverticular pain?]* Yeah, it's, well, it's a normal thing when I overdo it with fibre***.*** *[Okay, so it's normal for you?]* Yes. *[Right, so you don't think that's related to the Atezo?]* Maybe it's, maybe it's made me more sensitive, but I don't think so. [And apart from that, how have you felt?] A bit tired. (0012) | |

**Supplementary table 3. Quotes relating to Major Theme Causality**

| **Uncertainty on the part of clinicians** | | *“And did anybody say that this could be, you know, due to the Pembro?”*  “Well, the team did eventually say, "Well, we've pretty much eliminated everything else, it has to be a reaction to Pembrolizumab, you have no signs of infection or...". (0002) |
| --- | --- | --- |
|  |  | “Dr X said he’s still worried whether the diarrhoea is the radiotherapy or it’s the immunotherapy”. (0004) |
|  |  | “Dr X he said he doesn’t think it’s [back pain] anything to do with the immunotherapy but he said just keep an eye on it, you know”. (0010) |
| **Justification of symptoms or assumptions by patients** | Symptom related to cancer | “I think the breathlessness is, it's more linked to, I think, the progression of the disease”. (0005) |
|  |  | “I didn’t have much appetite and I think that was a cancer-related thing rather than a treatment-related thing” (0009) |
|  | Symptom(s) hangover effect from prior treatment | “My nails are horrible, but I'm thinking this is because of the chemo, I don't know, because they're growing out, you see. Can you see where it's all horrible on the ends of them, gross, and they keep splitting and breaking. But the new bit isn't like that, so I'm wondering whether that's just a case of the, if that's from the chemo”. (0007) |
|  | Symptom(s) related to other medication | “I still suffer with fatigue, I’ve suffered with that ever since I had been taking tablets for an irregular heartbeat. Even though there’s not a lot wrong with me”. (0001) |
|  | Psychological side effects | “… I had cycle one and then three weeks later I went for my appointment and I said, he said “how do you feel?” I said “I feel fine”, so had another treatment and then I don’t know, maybe it was before cycle three I said “I feel like I’m breathless, I’m short of breath more than I was”….Dr X thought about it long and hard, he said “one of the more challenging side-effects of this is pneumonitis, I’m going to do a scan for pneumonitis”….and so we did a scan after the fourth cycle and the one thing I would say is immediately after I was told I didn’t have pneumonitis the whole idea of shortness of breath had gone”. (0003) |
|  | Personalisation – “normalisation” of symptoms by patients | “I’m taking tablets anyway so I do feel tired normally but when I started out I was extra tired”. (0001)  “I think it was related to the treatment because I don’t usually suffer from rashes”. (0001) |
|  |  | “No, it's painful, more like gas. *[And is it like your normal diverticular pain***?*]*** Yeah, it's, well, it's a normal thing when I overdo it with fibre. **Okay, so it's normal for you?** Yes.  **Right, so you don't think that's related to the Atezo?** Maybe it's, maybe it's made me more sensitive, but I don't think so. My opinion is that it's not the medicine, it's me”. ((0012) |
|  | Other assumptions | “I never actually gave it a thought that it (the diarrhoea) was anything to do with the treatment. *[No, because you’re on holiday].* My first thought was the water or the salad or something in the hotel”. (0004)  “I thought pneumonitis would exclude me from having immunotherapy”. (0003) |

**Supplementary table 4. Quotes relating to Major Theme Decision making**

| **Reporting a potential ir-AE by patients** | How reported | “So I phoned up my CNS and I, I don’t know if I had an appointment that day, but I came up to the clinic and saw, it wasn't an oncologist, I saw this other doctor who was like a GP ...  *Did you, you waited till you came up to the clinic, then, you didn't try and call anybody from home, you know...?*  Well, I did, I phoned up the CNS nurse at the clinic...*And she said, "Come in"?*  Yeah, and she said to come up, and so that's what I did. (0007) |
| --- | --- | --- |
|  |  | *“And have you mentioned this [bowel pain] to anybody…*No, no. [*…you're going to today?]* Yes, today”. (0012) |
|  |  | *“Okay, when you said you let the doctor know, was that at your next clinical appointment or did you ring up and say you had a rash?*  No, no, I told them at the clinical appointment”. (0001) |
|  | Factors affecting method of reporting | “Because, apart from the inability to walk and the fact that I wasn't eating that much, I wasn't actually feeling, I was coughing a little bit, but I wasn't feeling that unwell, and I thought, you know what, I'll wait until I see the people that know what they're talking about rather than having to go through the A&E system again. ….  I discovered that if you phone out-of-hours, you don't necessarily get to know, get to talk to someone that knows anything about what you're talking about anyway, I found that out with my wife when she was having chemo, etc, etc, I did that a couple of times, had to phone out-of-hours, and in general, they're going, "Oh, I don't know, can you phone back in the morning". (0002) |
|  | Length of time to report | *“I mean, do you think you left it too long to report it?* No, not necessarily, because it was only ten days”. (0002) |
|  |  | *“So when you said that you developed this really serious rash, and you didn’t call… how long did you wait, how long was the gap between that and your next appointment, I mean are we talking days or a week or…?*  Yeah, I’m trying to think back a week or two, a week or so, you could say two weeks at a maximum”. (0006) |
|  | Factors affecting length of time to report | “And then it was the bank holiday weekend, so there wasn't a clinic anyway, but I had a regular Monday scheduled clinic the following week, which I think was about the 3rd of September, and I thought, well, I'm surviving okay at home, I haven't got anything specific that I have to do, so I'll hang on until clinic day and hobble up there then and throw myself at their mercy.  *Right, okay, so this was a period of days before your next clinic?* It was a week”. (0002) |
| **Management of a potential ir-AE by patients** | Self-medication | *“Right, okay, and did you do anything about the rash, did you go and get any cream yourself?*  I got some cream and I put it on to it…Just ordinary cream you know, that stops kind of an itchy rash. (0001) |
|  |  | “Because my knee's starting to swell I've started to take some Naproxen which I had left over from the last time”. (0002) |
|  |  | “We had some Imodium, one of those things we always take, and that didn’t make any difference at all but then I started to think well perhaps it is something to do with the treatment or the drugs because I’m on quite a few different painkillers.  *And did you remember the information?* I did but because, because I had been so well after the others it was sort of down the list of things and as it happened when we was packing I found a box of diarrhoea tablets that they gave me when I was having the chemo way back and I just threw them in the case thinking oh they might be useful! So I took those and again they didn’t make any difference and then when I sat down and read what was in the prescribed diarrhoea tablets and what was in the Imodium it was exactly the same”. (0004) |
|  |  | *“And did you go and get them [immodium] from the chemist?*  Yeah. And…*[And how did you use them, just afterwards or preventatively?]* When needed. Usually straight afterwards. (0011) |
|  | Consulting others/ internet | “My daughter is involved at some levels in the pharmaceutical industry and she came back quite quickly saying at least 20% of the patients on immunotherapy do suffer this thing (rash)”. (0006) |
|  | Other strategies | **“**If I'm feeling breathless, when I say feeling breathless, it’s after climbing a flight of stairs or whatever, you know, so if I'm breathless after climbing the stairs, I'll just stay upstairs for a bit, it's not a problem”. (0002) |
| **Management of a potential ir-AE by clinicians** | Medication | “I saw this other doctor who was like a GP pain.. yeah, and she had a look, and obviously it was, she could see it was quite swollen, so she went off and had a chat with somebody else, and then they had a look at the screen and what have you, and then I think they came back and said, "We think it is the Pembro", so they just gave me the steroids to try and take the swelling down and everything and just to sort of monitor it. *[You were given some steroids and you say that that improved it within a week?]* Yes, within a week it went down”. (0007) |
|  | Input from specialists/ liaising with other hospitals | “I had pericardial effusis *[sic]*. ….They drained 150ml of fluid out of my heart, well out of the pericardium, and it was like they flicked a switch, I went from being almost dead to being very hungry. [*Within, are we talking within a 24 hour period once they gave you this treatment?]* once I could actually breathe again it was, you know, it was literally like they'd flicked a switch, once I could breathe properly and, you know, my systems were working again, everything just clicked back into place. ….So the rheumatology team, who'd been drafted in by the oncologists, drained fluid out of that one as well”. (0002) |
|  | Sending patient to A&E | “I actually virtually crashed in the Clinic.. And they looked after me until they could get me round to A&E, who looked after me some more while they all decided what was wrong with me, a big committee of doctors surrounding me in resus in A&E, and eventually at about eleven o'clock at night they threw me in an ambulance and shoot me off to hospital”. (0002) |
|  | Hospitalisation | "Oh, we're going to ring your GP, see if they can sort of come round and have a look at you". [*And the GP said you had an infection?]*  Yeah, and he came round and examined me and said, "Oh, I don't like the sound of what's going on in your chest", all these crackling noises, and they just say, "Okay, that's it, call an ambulance, off you go. [*And so you were admitted to hospital, were you?]* Yes, yeah. (0005) |

**Supplementary table 5 Quotes relating to Major Theme IMPACT**

| **Impact of IO treatment** | Physical | *“No, and it hasn’t had any impact on your life, you’re still able to do all the things that you know, you were able to do before starting the treatment…*Yeah, yeah, yeah, yeah, I’m out and about every day, I might be a bit slow but I can manage to get around, go on the buses and do my shopping and stuff like that”. (0001) |
| --- | --- | --- |
|  |  | *“Has it had any impact on you sort of in the sense of, has it made you do things differently in your life..?* No, although I might stick to this country for a while until I know exactly what's going on….”. (0002) |
|  |  | *“So the impact of the Pembro overall has been a positive experience for you?* Absolutely. There’s no negatives for me. *[There’s no negatives at all?]* Bit of fatigue”. (0003) |
|  |  | “I have been, the last couple of times I've been down I've come by ambulance, because after I was in hospital I just got too weak, I didn't feel safe on the train, you know, I was getting too breathless around the house and what have you, sort of thing, I just thought, no, I’m not going to take, if I got pushed over, you know, I would fall over”. (0005) |
|  |  | “It’s the easiest one he has. Really, he doesn’t feel anything. Chemo, he used to feel dizzy, sick. Radiotherapy he still felt tired, he wasn’t sick but he was tired. This one, he doesn’t feel anything”. (0013) |
|  |  | “..this seems to have worked really very well with me because I went from feeling drained and fed up and everything after the radio-therapy to feeling much, much better….***[****so you say you’ve had the fourth round and you started to say you’ve immediately felt better]* I immediately felt better after I started it, so that’s a couple of months ago. [*Right, OK, and better in what way?*, I felt less tired, had more energy and in the early days of the cancer diagnosis I didn’t have much appetite and I think that was a cancer-related thing rather than a treatment-related thing. Anyway, when I’d stopped the radiotherapy, gradually the cancer, um, the appetite came back”. (0009) |
|  | Psychological | *“How did you feel at the beginning when they went through this treatment …?* Scared”. …  “The only thing is I know I’ve got to have a scan, then the old brain starts going……[*So anxiety around the scan time?]* Yes. Anxiety, yes. That’s the word, yeah”. (0011) |
|  |  | “It was a concern that I don't want to end up like I was before, and of course if I'm not on the treatment, will I start deteriorating, because I do know this was, more or less, a last chance, basically, there wasn't much else they could do for me. So it's a kind of a double-edged sword that, to be on it and to be so well is fabulous, but the other side, you know, in the back of your head was a little, every now and then a little voice says, "Aha, but when it finishes..." (0007) |
|  |  | The first cycle, it was, you know, the insecurity of how it's going to *[laughs]* that was... [*What, whether it was going to work or what might happen?]* Yeah, yeah, that was a psychological thing that, it was personal, you know, how is it going to work and all that”. (0012) |
| **Impact of reporting or experiencing an ir-AE** | Physical | “Well actually flying home was a real worry because I thought if I have, you know, an attack [of diarrhoea], for want of a better description, on the plane”. (0004) |
|  | Psychological | “I feel a bit disappointed because what Dr X has said to me today is that it’s more than likely that I’d have to come off the immuno-therapy and go onto a different type of chemotherapy because me body’s not tolerating it because I think the immunotherapy can go on for up to 2 years”. (0004) |
|  |  | *“*If they say, at some point, "You can't have it any more because it's doing you more harm than good", then quite what plan B will be, I don't know, and I don't really want to think about plan B until plan A is no longer an option”. (0002) |
